# High-Throughput Screen for Identifying Small Molecules That Target Fungal Zinc Homeostasis

**DOI:** 10.1371/journal.pone.0025136

**Published:** 2011-09-29

**Authors:** Claudia Simm, Chi-Hao Luan, Eric Weiss, Thomas O'Halloran

**Affiliations:** 1 Chemistry of Life Processes Institute, Northwestern University, Evanston, Illinois, United States of America; 2 Department of Molecular Biosciences, Northwestern University, Evanston, Illinois, United States of America; 3 Department of Chemistry, Northwestern University, Evanston, Illinois, United States of America; Research Institute for Children and the Louisiana State University Health Sciences Center, United States of America

## Abstract

Resistance to traditional antifungal drugs has increased significantly over the past three decades, making identification of novel antifungal agents and new targets an emerging priority. Based on the extraordinary zinc requirement of several fungal pathogens and their well-established sensitivity to zinc deprivation, we developed an efficient cell-based screen to identify new antifungal drugs that target the zinc homeostasis machinery. The screen is based on the zinc-regulated transcription factor Zap1 of *Saccharomyces cerevisiae*, which regulates transcription of genes like the high-affinity zinc transporter *ZRT1*. We generated a genetically modified strain of *S. cerevisae* that reports intracellular zinc deficiency by placing the coding sequence of green fluorescent protein (GFP) under the control of the Zap1-regulated *ZRT1* promoter. After showing that the GFP fluorescence signal correlates with low intracellular zinc concentrations in this strain, a protocol was developed for screening small-molecule libraries for compounds that induce Zap1-dependent GFP expression. Comparison of control compounds and known modulators of metal metabolism from the library reveals a robust screen (Z′ = 0.74) and validates this approach to the discovery of new classes of antifungal compounds that interfere with the intracellular zinc homeostasis. Given that growth of many pathogenic organisms is significantly impaired by zinc limitation; these results identify new types of antifungal drugs that target critical nutrient acquisition pathways.

## Introduction

*Candida* species are pathogenic organisms that account for most fungal infections in humans [1], [2]. With emerging drug resistance these pathogens have become a serious health problem, especially in immune-compromised patients with human immunodeficiency virus (HIV), chemotherapy patients, or transplant recipients [3], [4]. Despite intensive research only three types of antifungal drugs are currently in clinical use for systemic fungal infections. The first class targets enzymes of the ergosterol biosynthesis pathway. Ergosterol is the major sterol of the fungal plasma membrane and is important for its fluidity and integrity and therefore for the proper function of membrane-bound enzymes [5]. The second class, Echinocandins, is used in severe cases of Candidiasis. They inhibit β-glucan synthase and therefore interfere with cell wall biosynthesis [6]. The last class of antifungal drugs are nucleoside analogues such as 5-Flucytosine. This compound is imported into the cell and modified like cytosine, which results in production of 5-fluoro-UMP, a DNA synthesis inhibitor, and 5-fluoro-UTP, which disrupts protein synthesis when incorporated into RNA. However, the increasing resistance to these antifungal drugs coupled with the increasing number of immune compromised and AIDS patients makes it important to identify novel antifungal agents and new targets. Since pathogenic organisms require extraordinary concentrations of zinc and are highly susceptible to zinc deprivation, we have turned our attention to the high affinity pathways involved in zinc acquisition and utilization.

Prior studies have shown that zinc is an essential micronutrient for the proliferation of pathogenic organisms [7], [8], [9]. In fact, many organisms are more susceptible to loss of zinc than to iron deprivation [10]. The intracellular demand for zinc is quite high within bacterial and eukaryotic cells, which maintain zinc concentrations in the millimolar range. Despite this high total intracellular zinc concentration, very few zinc ions are free or weakly bound within the cell [11]. In eukaryotic cells those millimolar concentrations of zinc ions are often found in compartment, e.g. vacuoles [12], while concentration of free zinc in the cytosol is estimated to be in the lower nanomolar range [13]. Moreover, several zinc transporters have been reported as virulence factors in gram-negative and gram-positive bacteria as well as eukaryotic pathogens [8], [14], [15], [16]. Zinc transporter knock-out mutants are no longer able to grow in a low zinc environment and show reduced virulence. Given these observations, zinc homeostasis pathways show promise as target for antifungal drugs.

Studies of the budding yeast *Saccharomyces cerevisiae* show that zinc import, export, and redistribution are tightly regulated to maintain a relatively constant intracellular zinc level [17]. The zinc activated protein 1 (Zap1) is one factor that modulates expression of gene products involved in zinc trafficking, such as the zinc uptake transporter. Zap1 is a transcription factor that acts by binding to zinc responsive elements (ZRE) of the promoter regions of high affinity zinc regulated genes at high intracellular zinc, and thus leading to the inhibition of gene transcription [18], [19]. If intracellular zinc level drop Zap1 loses its bound zinc and transcription occurs. Recently, Zap1 orthologs have been reported in *Candida albicans* and *Aspergillus fumigatus* suggesting that similar zinc regulation circuits exist in these pathogenic fungi [20], [21].

We describe here the development of a screen for new antifungal agents that interfere with zinc homeostasis. To accomplish this, we created a promoter-reporter construct by fusing a 1 kb promoter fragment containing ZREs of the *S. cerevisiae* high affinity zinc transporter *ZRT1* to the sequence of green fluorescent protein (GFP). GFP is used in various biochemical applications like protein tagging, monitoring of gene expression, imagine of protein-protein interaction but recently also for drug screening purposes [22], [23]. Successful screens for anti-HIV drugs and chemopreventive agents using the promoter-GFP strategy have been described, indicating that the proposed zinc sensor might be able to detect new antifungal drugs targeting zinc homeostasis proteins [24], [25]. In this study we successfully developed a zinc specific high-throughput drug screen. Our analysis identified several compounds that interfere with zinc homeostasis. Surprisingly, some of them did not alter total zinc concentration but are likely to target intracellular sequestration and distribution pathways.

## Results

### Yeast transformants report zinc status

**Figure 1:**
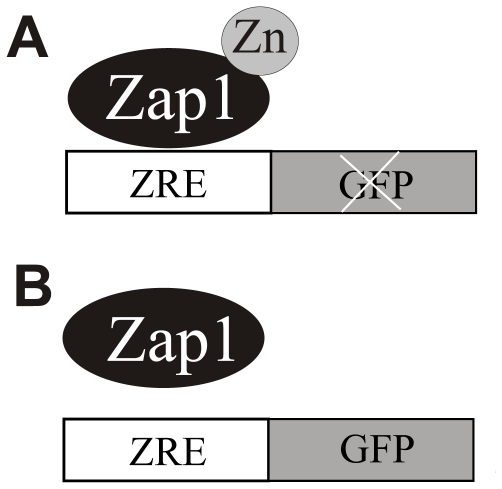
Model of zinc-regulated gene expression. A) Under high or normal zinc conditions Zap1 binds zinc and blocks the ZRE elements of the *ZRT1* promoter and therefore inhibits GFP expression. B) Under zinc deficient conditions Zap1 releases zinc then disassociates from the *ZRT1* promoter, which leads to the expression of GFP.

In order to develop a high-throughput screen for agents that disrupt zinc homeostasis we generated a yeast strain that expresses GFP specifically under conditions of zinc deprivation. We accomplished this by fusing the sequence of the promoter region of the *ZRT1* transporter gene with GFP. Previous studies have shown that the Zap1 protein suppresses zinc-responsive gene expression by binding to ZRE elements in target promoters [17], [18]. Therefore, genetically modified yeast cells expressing the ZRE-GFP promoter-reporter construct serve as a zinc sensor system. Under high and normal zinc concentrations GFP expression is inhibited by Zap1 (Figure 1A). When zinc concentration drops below a physiologically sufficient level GFP expression occurs (Figure 1B).

**Figure 2:**
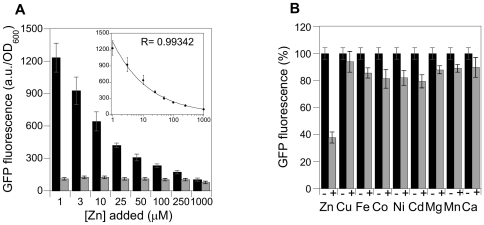
Zinc responsiveness of ZRE-GFP promoter-reporter construct. A) Comparison of Zn-dependent GFP fluorescence in untransformed yeast cells (gray bars) and yeast transformed with the ZRE-GFP promoter-reporter construct (black bars). Cells were grown in LZM medium with indicated zinc concentrations. GFP fluorescence and cell density were measured after 20 hours of incubation at 30°C. The inset shows a power law fit through the data. The excellent quality of the fit (the linear correlation coefficient between the fluorescence and the zinc concentration to the power −0.372 is R = 0.993) indicates that the fluorescence decreases with increasing zinc concentration in a scale-free manner. B) Metal specificity of the promoter-reporter construct. Transformed cells were grown in LZM either without (black bars) or with 100 µM (gray bars) of indicated metal ions and analyzed as before. GFP fluorescence is expressed as percent of non-supplemented control. The fluorescence signal was always normalized by cell density. Error bars represent standard deviation (SD).

We avoided plasmid copy number effects associated with episomal expression by integrating the ZRE-GFP promoter-reporter construct into the genome of the *S. cerevisiae* strain DY1457. The response of this sensor strain to zinc deficiency was then evaluated by growing cells in LZM medium containing a range of zinc concentrations (1 µM to 1 mM). As expected, expression of GFP was activated under low zinc conditions in cells containing the zinc sensor construct (Figure 2A). We found that GFP fluorescence intensity decreased logarithmically with increasing zinc concentrations in the growth medium (Figure 2A inset). The maximal GFP induction measured was 12-fold at 1 µM zinc supplementation. No GFP fluorescence was observed in samples grown in medium with 1 mM Zn. The fluorescence signal at this condition is comparable to autofluorescence generated by untransformed DY1457 cells.

### Zinc sensor construct is specifically responsive to zinc and not to other metals or cellular stress

In order to demonstrate that the promoter-reporter construct only responses to changes in zinc concentration but not to other metal ions we added solutions of 100 µM of zinc or various other divalent metal ions to LZM already supplemented with 10 µM Zn. As shown in Figure 2B, only the addition of zinc to the medium results in a significant decrease in GFP expression while only small changes in GFP fluorescence was observed with other metal ions. This suggests metal specificity of this method.

We also investigated the responsiveness of the sensor to other cellular stress conditions like heat and oxidative stress. Neither heat treatment nor the addition of H_2_O_2_ resulted in an increase in GFP expression (Figure S1). Moreover additional insights about oxidative stress response were obtained from screening the small-molecule Spectrum library. Redox active compounds included in the library, like menadione, benzoyl peroxide or hydroquinone, have been shown to generate reactive oxygen species (ROS); however they do not increase GFP expression.

### GFP expression increases after treatment with proof-of-concept compounds

**Figure 3:**
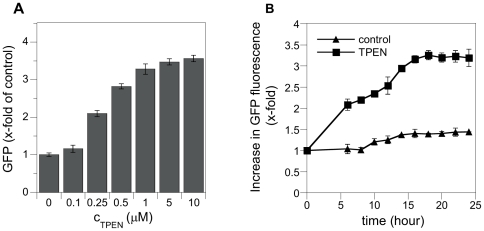
Dose and time response of GFP fluorescence after treatment with TPEN. A) Cells transformed with the ZRE-GFP promoter-reporter construct were grown in RPMI-1640 medium with indicated TPEN concentrations for 20 hours at 30°C. Fluorescence signal was normalized by cell density and is expressed as x-fold increase of untreated sample. B) Cells containing the ZRE-GFP promoter-reporter construct were grown in absence or presence of 5 µM TPEN in RPMI-1640 medium at 30°C. Fluorescence and cell density were measured at times indicated. Fluorescence signal is expressed as x-fold increase of the 0 hour time point. Error bars represent standard deviation (SD).

Next, we tested the performance of the zinc responsive reporter strain with compounds known to interfere with zinc homeostasis, including metal chelators. As expected, treatment with chelators like EDTA, DTPA (Figure S2), and TPEN (Figure 3A) resulted in increased GFP expression in a dose-dependent manner. Because EDTA and DTPA are not cell permeable, this unsurprisingly demonstrates that chelation of zinc in the extracellular medium renders the metal unavailable for the cell and results in decreased intracellular zinc concentration. TPEN, on the other hand, has a higher affinity for zinc (log K = 15.2 at pH 7.6) and can scavenge zinc in the external medium as well as inside the cell [11]. It was the most effective inducer of GFP expression among the proof-of-concept compounds. A significant increase in GFP fluorescence was observed at TPEN concentrations as low as 250 nM. GFP expression further increased at higher concentration of TPEN reaching a maximum at 5 µM. Thus, we used TPEN concentration of 5 µM as positive control in our efforts to identify compounds that disrupt zinc homeostasis.

### GFP expression is time-dependent

Previous studies have shown that zinc accumulated in the yeast vacuole can sustain growth for 6 to 8 doublings [12]. Since zinc concentrations have to be sufficiently reduced in order to trigger an increase in GFP expression, determining the optimal assay time point was crucial. Therefore we treated cells with 5 µM TPEN and compared GFP fluorescence and cell density relative to untreated control every 2 hours. After 6 hours of incubation in 5 µM TPEN, we observed a 2-fold increase in fluorescence. The maximum in GFP fluorescence for TPEN treated cells corresponded to a 3-fold increase reached at 18 hours of incubation (Figure 3B). Over this time, increase in GFP expression in control cells was negligible.

The standard Z′ factor and signal to noise ratio for each time point provide a direct evaluation of the robustness of this reporter system for screening purposes (Table 1). Following established approaches, the Z′ factor is defined as Z′ = 1−((3σ_+_+3σ_−_)/(μ_+_−μ_−_)) [26]. In this study, σ_+_ and σ_−_ are the standard deviation of GFP signal in positive control (TPEN) and negative control (untreated sample), and μ_+_ and μ_−_ are the mean positive and negative control signal. Most Z′ values shown in Table 1 were above 0.5. The Z′ value of greater than 0.5 indicates that the ZRE-GFP promoter-reporter construct is suitable for large scale compound screening. The second assay robustness criteria is the signal/noise ratio, which is defined as S/N = (μ_+_−μ_−_)/((σ_+_)^2^+(σ_−_)^2^)^1/2^ [27]. Numbers for this indicator should be high. For most time points their values are between 10 and 17, demonstrating again that this is an excellent assay. According to these criteria time points of 18 and 20 hours were optimal. We therefore measured GFP fluorescence and cell density after 20 hours for all high-throughput screens.

**Table 1:** Summary of robustness criteria of the promoter-reporter assay.

Time point (h)	TPEN GFP vs. untreated control GFP (x-fold)	Z′ value	Signal/Noise
6	2.01	0.3	5.99
8	2.18	0.74	16.07
10	1.95	0.67	12.65
12	2.03	0.31	5.75
14	2.15	0.91	35.88
16	2.25	0.68	13.24
18	2.34	0.79	17.06
20	2.28	0.74	15.19
22	2.25	0.61	10.75
24	2.22	0.65	8.68

### Screening of a small-molecule library

**Figure 4:**
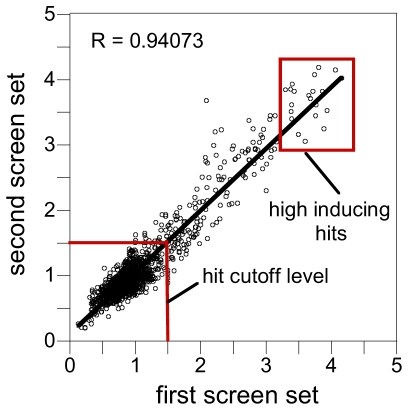
Screen results are reproducible. The GFP intensities of replica plate sets of screen 1 and screen 2 were plotted against each other. The linear regression between both plate sets demonstrates reproducibility of hit compounds. The cut off level for hit selection is 1.5-fold. The red box shows hits with high GFP induction level.

**Figure 5:**
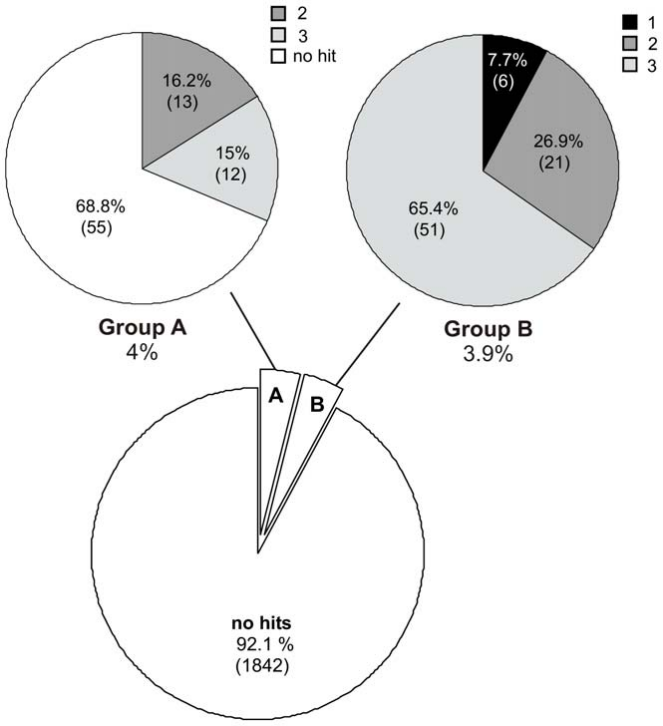
Summary of screen results. 3.9% of all screened compounds showed GFP induction at 100 µM concentration (group B). Compounds that proved cytotoxic at 100 µM (4%) were serially diluted and reanalyzed (group A). Positive hits of both groups were categorized into classes according to their degree of GFP induction. Category 1 hits showed induction of 80% and higher. For categories 2 and 3 GFP induction was 50%–80% and 30%–50%, respectively. The percentage and number of compounds (in brackets) are shown for each class and category.

Having defined optimal assay conditions for the ZRE-GFP promoter-reporter construct, we screened the Spectrum Collection library, a small-molecule library of 2000 compounds. These compounds were preselected to cover a wide range of biological activities and structural features. Half of the compounds are drugs with defined pharmacological and toxicological profiles, and the remainder are natural products (30%) and bioactive compounds like enzyme inhibitors or receptor blockers (20%). We assayed compounds in duplicate at a single concentration of 100 µM. The reproducibility of the screen is shown in Figure 4, where hit correlation between two separate screen plates is linear (R = 0.94) indicating that the screen results were highly reproducible. We defined compounds that induced GFP expression more than 1.5-fold or 30%, respectively, of negative control as candidates for further analysis. Approximately 4% of the compounds (80 compounds) inhibited growth completely at 100 µM and were serially diluted and rescreened in two-fold dilutions steps between 100 µM and 0.19 µM. We refer to this set as Group A (Figure 5). After examining ZRE-GFP induction at lower compound concentrations, we found that only one third of these compounds meet the criteria of being a hit. This indicates that cell toxicity in general does not trigger ZRE-GFP expression and is consistent with our earlier finding that other cellular stresses do not induce Zap1-dependent GFP expression. GFP-inducing compounds that allowed growth at 100 µM are classified as Group B. In total there were 103 reproducible hits (5.15%) with 78 hits (3.9%) derived from Group B and another 25 (1.25%) from Group A. A list of all compounds identified can be found in Table S1. We organized compounds of both groups in 3 categories according to the magnitude of GFP induction in comparison to negative control (category 1: >80%, category 2: 50–80% and category 3: 30–50%). All compounds of group A induced GFP expression between 30–80% only. Taken both groups A and B together a total of 34 and 63 compounds were found in the categories 2 and 3, respectively. Six category 1 compounds with high GFP fluorescence were found in Group B.

### Validation of selected hit compounds

**Figure 6:**
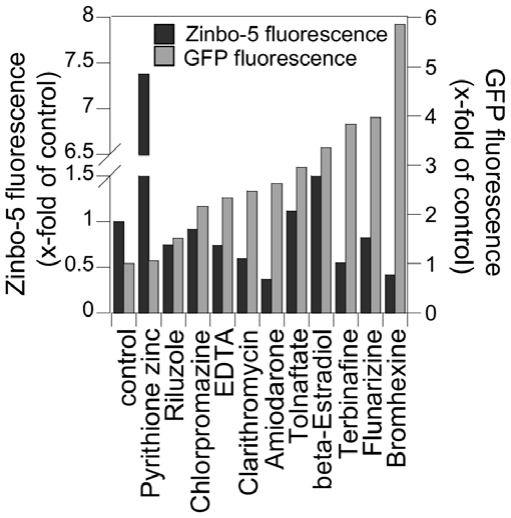
Validation of selected hits. A subset of hit compounds was subjected to flow cytometry analysis. Cells were treated with hit compounds and incubated for 20 hours at 30°C. Labile zinc content was measured using the zinc fluorescent probe Zinbo-5. Zinbo-5 fluorescence was plotted in comparison to GFP fluorescence.

Even though stress or cell toxicity did not affect GFP expression we wanted to determine if other secondary effects could trigger an increase in GFP signal. Therefore, we developed a series of validation experiments for commercial available compounds in the top 30% of categories 1 and 2 in group B hits. In order to determine if increased ZRE-GFP expression was indeed caused by a decrease in available intracellular zinc we treated cells with selected primary hit compounds and stained them with the zinc fluorescent probe Zinbo-5. The affinity of this probe for zinc (K_d_ = 2.2*10^−9^ M) is lower than that of most zinc-binding proteins, and thus it only reports the free or weakly bound zinc ions [28]. We then analyzed Zinbo-5 stained cells by flow cytometry where dead cells were identified by propidium iodide staining and excluded from the assay. In this analysis, Zinbo-5 fluorescence emission at 463 nm is indicative of labile zinc ions. As control compounds, we analyzed the effects of the metal chelator EDTA and the ionophore Zn-pyrithione. EDTA binds zinc with a high affinity (log K = 14.3) [29] and growth in EDTA-containing medium (500 nM) leads to significantly lower accumulation of intracellular zinc (0.9 nmol/mg dry weight). This corresponds to a 50% reduction relative zinc content of cells grown in the absence of EDTA (1.76 nmol/mg dry weight), whereas growth in medium containing Zn-pyrithione increases intracellular zinc level [30]. As expected, we found that EDTA treatment resulted in low Zinbo-5 fluorescence but high GFP fluorescence, while Zn-pyrithione treatment led to high Zinbo-5 fluorescence and low GFP fluorescence (Figure 6).

We found that the intensity of Zinbo-5 and GFP fluorescence were inversely related for most compounds tested. For these compounds, we therefore conclude that the observed increased ZRE-GFP expression can be explained by a decrease in labile intracellular zinc. Intriguingly, we found that tolnaftate and β-estradiol caused increased ZRE-GFP expression as well as Zinbo-5 fluorescence. Whereas no link between zinc homeostasis and tolnaftate is known, β-estradiol has been shown to alter zinc transporter activity and increase zinc plasma level [31], [32]. Although more compounds will be validated, the results to date are highly encouraging that most small molecules identified as hits in the high-throughput screen interfere indeed with zinc homeostasis.

### Analyzing identified drug leads in *Candida albicans*

**Figure 7:**
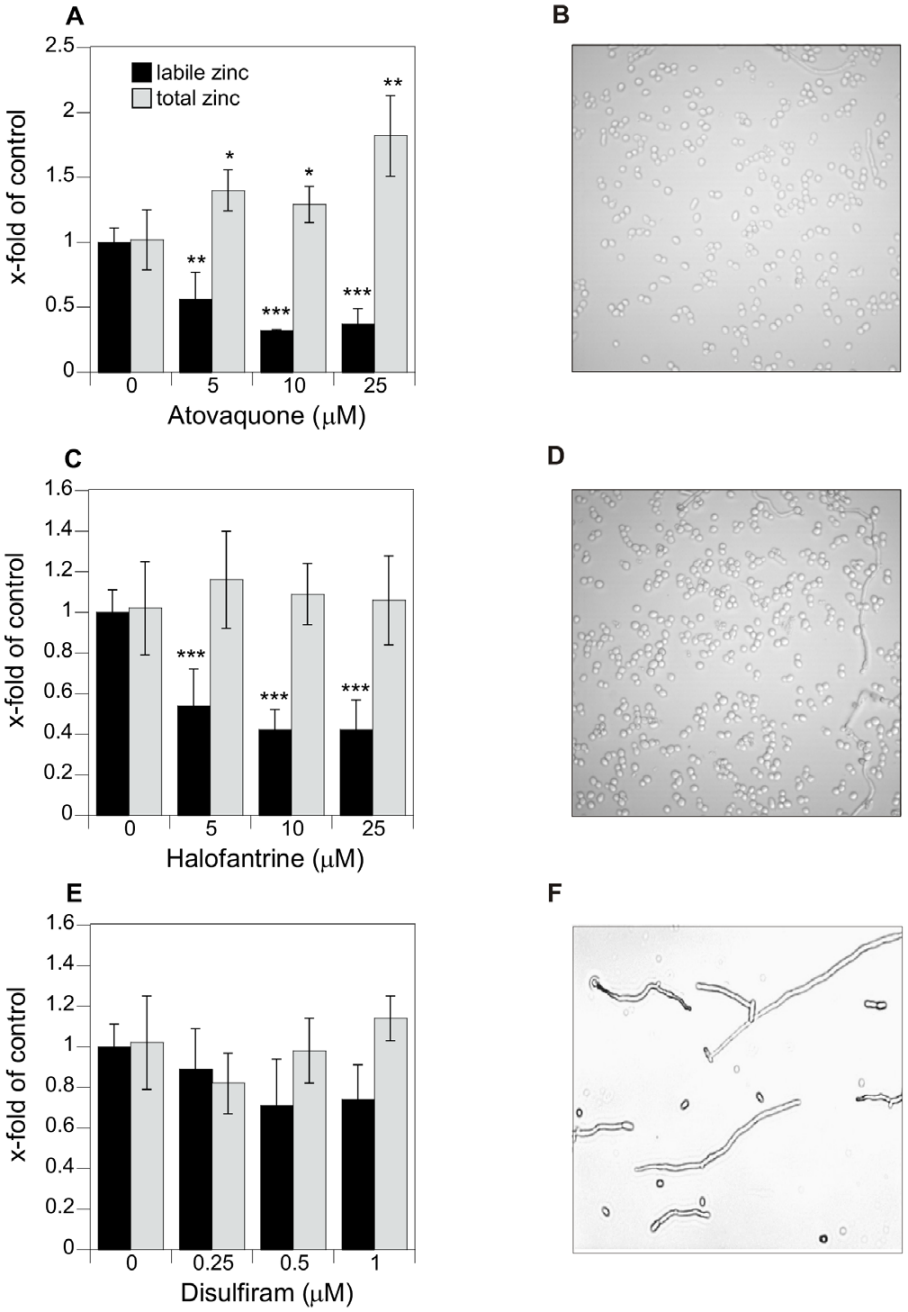
Total and labile zinc and cell morphology of *C. albicans*. *Candida* cells treated with A, B) Atovaquone, C, D) Halofantrine or E, F) Disulfiram and incubated at 30°C for 20 hours. 100 µl aliquots were immediately used for flow cytometry, the remaining culture was used for ICP-MS analysis. Light microscopy images were obtained from cells treated with 25 µM atavaquone and halofantrine and 1 µM disulfiram using standard protocols. Error bars represent standard deviation (SD). Level of significance are characterized as followed (* P<0.01, ** P<0.001, *** P<0.0001, not significant P>0.05). Statistical significance was tested by one-way ANOVA.

We have shown that the ZRE-GFP promoter-reporter construct was able to identify agents that interfere with zinc homeostasis in the non-pathogenic yeast *S. cerevisiae*. Next we evaluated the activity of the most promising hits in the pathogenic yeast *Candida albicans*. These agents were analyzed for dose dependent effects on cell viability, labile zinc (determined by Zinbo-5 as described above) and total zinc accumulation (measured by ICP-MS analysis). Since *Candida* is polymorphic yeast we also looked at its cell morphology after treatment with drug leads by light microscopy. Cell morphology, total and labile zinc content after atovaquone, halofantrine, and disulfiram treatment in *Candida* are shown in Figure 7. Atovaquone-treated *Candida* cells showed a striking increase in total zinc content but a decrease in labile zinc level (Figure 7A). These findings are consistent with an extreme disruption of zinc homeostasis. Li et al. reported a similar effect with clioquinol, a known antifungal agent. In that study, yeast treated with the clioquinol showed gene expression pattern of a metal deficient cell even though total metal ion levels were increased [33]. This was explained by a delivery of metals near the plasma membrane where they are unavailable for most metalloproteins.

Our results support the idea that zinc availability is generally limited in *Candida* and that atovaquone is a modulator of zinc homeostasis. Halofantrine also showed a decrease in labile zinc while total zinc concentration was unchanged (Figure 7C). Further increase in halofantrine concentration in *Candida* cultures resulted in total growth inhibition (data not shown).

Total and labile zinc concentrations seemed to be unchanged in *Candida* cells treated with disulfiram (Figure 7E) even though it is an inducer of ZRE-GFP expression. Whereas Candida cell treated with 25 µM atovaquone and halofantrine appear mainly in its yeast form (Figure 7B, D), disulfiram triggers hyphae formation in *Candida* (Figure 7F). This interferes with the consistency of flow cytometry data due to the infinite number of cell shapes and sizes. Therefore the demand for zinc in both forms could be significantly altered and the comparison of zinc concentrations in an untreated control in the yeast form with a disulfiram-treated sample in hyphae form might lead to artifacts.

## Discussion

In this study we describe the development and validation of a high-throughput screen for antifungal drugs targeting zinc homeostasis. The induction of a GFP signal driven by the promoter of a zinc-responsive gene in live *S. cerevisiae* cells provides a simple system to monitor changes in intracellular zinc availability. In contrast to many other high-throughput assays this is not a cytotoxicity screen. The assay not only reports on the ability of a candidate agent to inhibit growth, it also looks at a biological impact of that agent on key physiological pathways, namely those involved in zinc homeostasis. With proper controls and secondary assays, this dual index screen provides insights into mechanism of action of the hits. Furthermore, instead of measuring a decrease in zinc we analyze the amplification of a physiologically regulated fluorescence signal. Measuring a positive event abolishes artifacts or false positives caused by measuring too close to low sensitivity thresholds. This assay was optimized for high-throughput screens and due to its non-invasive nature is fast, easy, and inexpensive to perform. Moreover, this screen is not limited to an end point read-out. Since no staining or washing steps are required, it is compatible with time course experiments.

The differences in GFP signals between negative (untreated sample) and positive control (5 µM TPEN) resulted in a Z′ value of greater than 0.6. High sensitivity, good signal-to-noise ratio, and reproducibility further demonstrate the potential of this screen. The yeast-based live cell system selectively and sensitively responses to a variety of small molecule compounds that interfere with zinc homeostasis. As far as we are aware, this screen assay is the first designed to find hits throughout a metabolic pathway as opposed to a particular target protein. This increased the chances of identifying novel antifungals and new core structures.

Hits can be categorized according to their degree of GFP induction. Those with high GFP expression were put through validation tests using a small molecule fluorescent probe (i.e. the Zinbo-5 assay) to independently probe intracellular zinc availability in parallel with GFP expression [28]. A total of 80% of hit compounds tested showed an inverse correlation between GFP and Zinbo-5 fluorescence in *S. cerevisiae*, suggesting that in these cells, the ZRE-GFP promoter-reporter construct is able to identify compounds that interfere with zinc homeostasis. Zap1 has been shown to induce genes involved in oxidative stress or general cell stress response. However a zinc-independent Zap1 activation has so far not been described [34]. Moreover heat and oxidative stress do not increase GFP expression.

**Figure 8:**
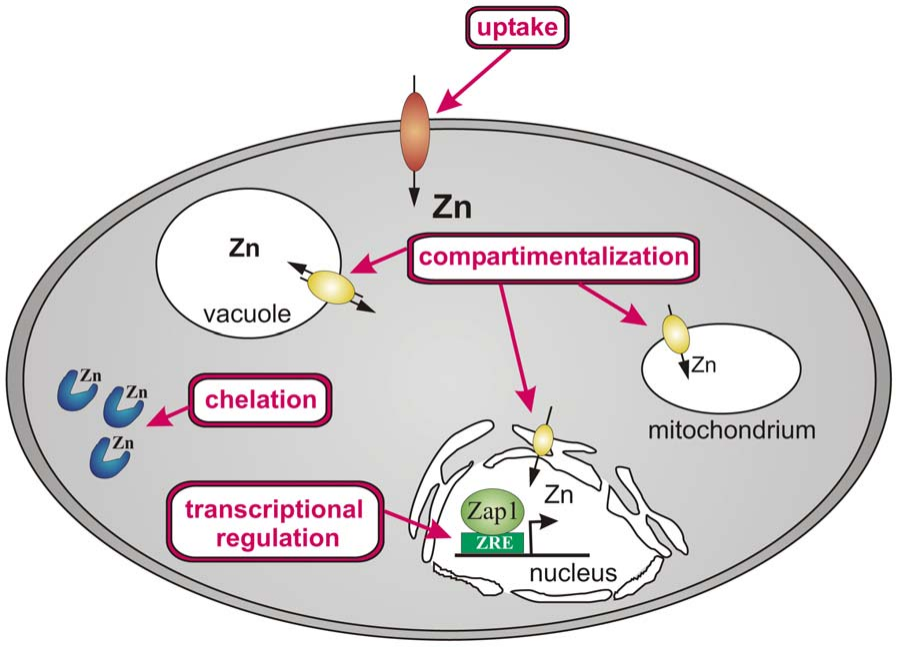
Summary of mode of action. The cell model shows the four potential mechanisms of action for hit compounds to interfere with zinc homeostasis. Hit compounds can inhibit zinc uptake transporter, induce the formation of chelators or act as chelators themselves, interfere with zinc transport in or out of compartments, or modulate zinc transcription factors like Zap1.

As for most cell based assays, this zinc reporter screen does not provide direct identification of the targets of these interesting new hits. Additional studies support the idea that antifungal drug leads identified in our zinc screen can target different mechanisms in zinc homeostasis (Figure 8). Some leads may work as zinc sequestering or chelating agents, which bind zinc in a form that prevents transport from the surrounding media into the cell, or they could bind intracellular zinc and make it unavailable to zinc-dependent metalloenzymes. The hits may also function as inhibitors of high affinity zinc uptake proteins or they could inhibit intracellular zinc trafficking and sensing proteins, i.e. vesicular zinc transporters, chaperones and transcription factors. The results described here are consistent with a model wherein atovaquone and halofantrine act as intracellular sequestering agents. They allow uptake of zinc into the cell but prevent access by zinc metalloproteins. Especially atovaquone might induce zinc deficiency perhaps by effectively interfering with zinc binding to Zap1, and therefore activating transcription of zinc uptake proteins and triggering an elevated zinc uptake from the external medium. Actual total and labile zinc level after disulfiram were more difficult to determine due to the morphological difference in comparison to untreated control cells. It is therefore not clear to which category of action mechanism this drug belongs. Most published data suggest that hyphae formation is triggered by zinc deficiency [35], [36], [37].

One additional concern may be the selective targeting of human rather than fungal proteins by hit compounds. To address this issue we performed extensive bioinformatic analysis to compare fungal and human Zip and CDF zinc transporter sequences. The results are summarized in Table S2 and Figure S3. In general, only little homology between fungal and human transporter sequences could be found. Moreover, we looked at conserved sequence motifs in both groups of zinc transporters. Whereas no common sequence motif could be found between fungal and human transporters of the Zip family, a HG/HS motif was shown in both fungal and human CDF zinc transporters. This functional motif, however, varies sufficiently in length and spacer amino acids that selective targeting is possible. Hit compounds that interact stronger with human proteins can easily be detected and eliminated by testing and comparing their toxicity between fungi and human cells; e.g. the effective concentration in *Candida* for atovaquone is 4 to 10 times lower than the approved maximal plasma concentration in human.

Taking all those observations in account, this zinc sensor screen system provides the first step in identifying new targets for antifungal agent that interfere with zinc homeostasis pathways. Coupled with other methods (e.g. confocal microscopy) this screen can identify mechanisms of action for lead compounds. We anticipate that results obtained in the *S. cerevisiae* screening system will apply to similar pathways in pathogenic yeast like *Candida albicans* and *Aspergillus spp*.

## Materials and Methods

### Strains and growth conditions

The *S. cerevisiae* strain used is DY1457 (*MATα ade6 can1 his3 leu2 trp1 ura3*). Yeast precultures were grown in YP medium plus 2% glucose (YPD) at 30°C. Transformants were plated on synthetic defined medium plus 2% glucose (SD) and histidine was omitted for plasmid selection. Initial GFP assays were carried out in low zinc medium (LZM) at 30°C [17]. GFP proof of concept experiments and high-throughput screens were performed at 30°C in RPMI-1640 medium without phenol red (R8755, Sigma-Aldrich, St. Louis, MO). Phenol red was omitted because it interfered with GFP reading. The medium was adjusted to pH 7.0. For heat shock experiments, cells were grown in RPMI-1640 medium to a cell density of 0.5 and incubation temperature was increase to 42°C for 10, 20 and 30 minute. Oxidative stress was applied by adding 0.1, 0.5 and 1 mM of H_2_O_2_ to yeast cultures at OD_600_ = 0.5. All cloning procedures were carried out in the *Escherichia coli* strain Top10F′ (Invitrogen, Carlsbad, CA). *E. coli* cells were grown in LB medium at 37°C with or without ampicillin. The *Candida albicans* strain 90028 was purchased from ATCC and grown in RPMI-1640 medium at 30°C for all experiments.

### Constructions of promoter-reporter construct

Standard molecular biology procedures were used for DNA manipulation and *E. coli* and yeast transformations [38], [39]. GFP sequence was amplified by polymerase chain reaction from phMGFP (Promega, Madison, WI) using the primer CGCG GGA TCC ATG GGC GTG ATC AAG CCC GAC and CGCG GCG GCC GC TTA GCC GGC CTG GCG GGG TAG containing a *BamHI* and *NotI* restriction site (underlined), respectively. For the amplification of the *ZRT1* promoter sequence genomic DNA of *S. cerevisiae* was extracted and used as template for PCR with the following primers CGCG CTC GAG TCA GAT ATT TCT TTT TTT GTC CCT and CGCG GGA TCC AAT TCC TTT TTT GAT ATT TGT GCT GTT containing a *XhoI* and *BamHI* restriction site. Both PCR products were digested with the appropriate restriction enzymes and ligated into the similarly digested plasmid pRS303 (ATCC, Manassas, VA) to fuse the *ZRT1* promoter with GFP. The resulting plasmid was linearized with *PstI* for integration at the chromosomal *HIS3* locus of DY1457. Transformants were plated on SD plates minus histidine for selection and positive transformants were selected for subsequent experiments.

### Validation of ZRE-GFP promoter-reporter assay

*S. cerevisiae* cells with integrated promoter-reporter construct were inoculated in LZM with different zinc concentrations at a starting cell density OD_600_ = 0.05. Cell suspension was transferred to 96-well black clear-bottom microplates (Costar, Cat. No 3603). After plates were filled they were sealed with AirPore tape (Qiagen, Cat. No. 19571) and incubated with vigorous shaking at 30°C. Following overnight incubation GFP fluorescence data (excitation 485/20 nm, emission 528/20 nm) were collected using an automated plate reader Synergy2 (BioTek). To correct for cell number variation in detection path due to non-uniform distribution of cells absorbance (OD_600_) was recorded for each well. The fluorescence read-out was then divided by the absorbance reading. All proof of concept experiments were performed in RPMI-1640 medium without phenol red. Compounds were dissolved in DMSO (Sigma-Aldrich, St. Louis, MO) and serially diluted. 2 µl of compound was pipetted to the wells of 96-well plates and 198 µl cell suspension adjusted to OD_600_ = 0.05 was added.

### High-throughput screen

The high-throughput screen was performed using liquid handling robots. A small-molecule library of 2000 compounds (Spectrum collection, MicroSource Discovery Systems, http://www.msdiscovery.com/spectrum.html) in DMSO (stock concentration 10 mM) was obtained. Two microliter of each solution were transferred to 96-well black clear-bottom microplates (Greiner, Cat No. 655090) using a Biomek FX 96 Multichannel robot (Beckman Coulter, Brea, CA). Immediately 200 µl cell suspension adjusted to OD_600_ = 0.05 in RPMI-1460 medium without phenol red was added to the plates using a Titan Stacker-Multidrop device (Titertek, Huntsville, AL). A negative (DMSO blank) and a positive control (5 µM TPEN) was included in each plate. Initial cell density and GFP fluorescence were measured after filling the plates. Plates were then sealed with Airpore tape (Qiagen, Cat. No. 19571) and incubated for 20 hours at 30°C in a HiGro shaker-incubator (GeneMachines, Genomic Solutions, San Carlos, CA). GFP fluorescence (excitation 485 nm, emission 528 nm) and cell density by OD at 600 nm were measured using a plate reader Analyst GT (Molecular Devices, Sunnyvale, CA). Compounds that proved to be excessively cytotoxic to yeast at the initial concentration of 100 µM were retested at a lower concentration. All compounds were serially diluted in DMSO using a Biomek FX Span-8 robot (Beckman Coulter, Brea, CA).

### Flow cytometry

Yeast cells from an overnight culture were adjusted to a density of OD_600_ = 0.05 in RPMI-1460 medium and treated with selected hit compounds identified in the high-throughput screen. After 20 hours of incubation at 30°C with shaking 100 µl aliquots of cell culture were transferred into flow cytometry tubes (Falcon, Cat No. 352054). 1 µl of a 1 mM stock solution of the zinc fluorescent probe Zinbo-5 was added and incubated for an additional 30 minutes at 30°C [28]. After diluting the cell suspension with 500 µl of PBS buffer 5 µl propidium iodide (500 µg/ml) was added for live/dead cell staining. Zinc fluorescence was then analyzed (excitation 358 nm, emission 463 nm) using the BD LSRII flow cytometer (Becton Dickinson, Franklin Lakes, NJ). Data for live/dead cell staining were obtained by excitation at 538 nm and emission at 617 nm.

### ICP-MS measurements

Cells were grown for 20 hours with a starting density of OD_600_ = 0.05 in RPMI-1460 medium with or without treatment. Cells were harvested by centrifugation at 1000× g for 5 minutes and then washed in ice-cold distilled deionized water. After cell pellet was frozen it was freeze-dried for several hours. The dry pellet was digested in 200 µl 65% HNO_3_ at 90°C for 30 minutes. The digest was filled up to a total volume of 5 ml with distilled deionized water and internal standard and immediately used for ICP-MS analysis (X-Series 2 ICP-MS, Thermo Fisher Scientific).

## Supporting Information

Figure S1 GFP expression after oxidative and heat stress treatment. Cells transformed with the ZRE-GFP promoter-reporter construct were grown in RPMI-1640 medium at 30°C to a cell density of 0.5. A) Oxidative stress was applied by adding 0.5 mM of H_2_O_2_. Cells treated with 5 µM TPEN were used as positive control. Cells continued to grow for 10, 30 and 60 minutes and GFP fluorescence and cell density was measured. B) Cells were quickly transferred from 30°C to a prewarmed 50 ml-falcon tube in a 42°C water bath. After cells were incubated at this temperature for 10, 20 and 30 minutes, GFP fluorescence and cell density was determined. Cells treated with 5 µM TPEN were used as positive control. Error bars represent standard deviation (SD).
(TIF)

Figure S2 Dose response of GFP fluorescence after treatment with DTPA and EDTA. Cells transformed with the ZRE-GFP promoter-reporter construct were grown in RPMI-1640 medium with indicated chelator concentrations for 20 hours at 30°C. Fluorescence signal was normalized by cell density and is expressed as x-fold increase of untreated sample. Error bars represent standard deviation (SD).
(TIF)

Figure S3 Motif comparison of Zinc transporters. Human and fungal zinc transporters of the Zip family (A) and CDF family (B) were analyzed for conserved functional motifs. Motifs of human CDF and Zip group I transporter are shown in red, hZip group II in blue and fungal transporters in green. TMpred and ClustalW2 were used for motif analysis.
(TIF)

Table S1 Summary of Hit Compounds.
(PDF)

Table S2 Protein sequence comparison between human and fungal A) Zip and B) CDF transporters.
(PDF)
