# Supplementary figures and images for: High-Throughput Screen for Identifying Small Molecules That Target Fungal Zinc Homeostasis
Source: PLoS One. 2011 Sep 29;6(9):e25136. doi: 10.1371/journal.pone.0025136 (PMC3182986; doi:10.1371/journal.pone.0025136)

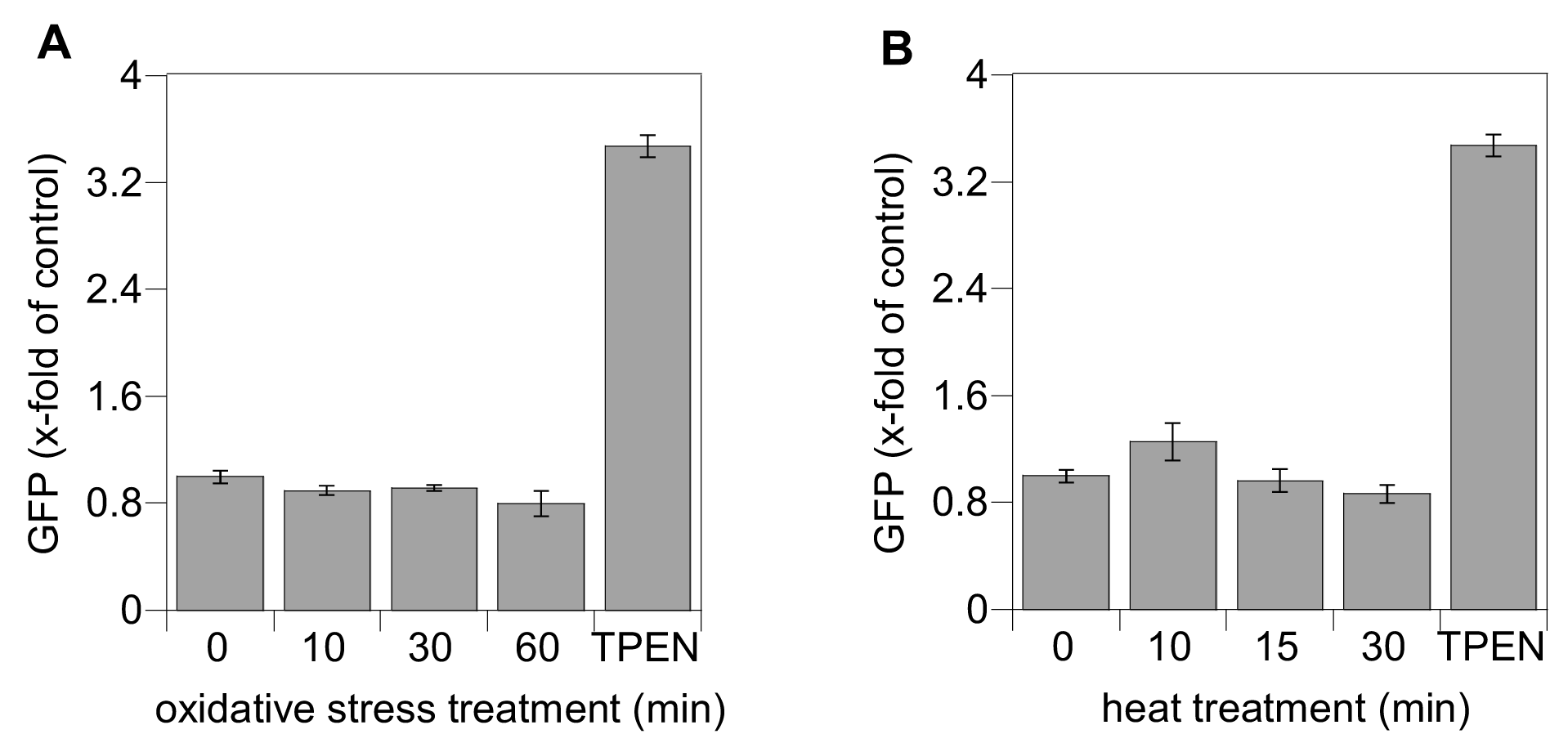

Supplement: Figure S1 — GFP expression after oxidative and heat stress treatment. Cells transformed with the ZRE-GFP promoter-reporter construct were grown in RPMI-1640 medium at 30°C to a cell density of 0.5. A) Oxidative stress was applied by adding 0.5 mM of H2O2. Cells treated with 5 µM TPEN were used as positive control. Cells continued to grow for 10, 30 and 60 minutes and GFP fluorescence and cell density was measured. B) Cells were quickly transferred from 30°C to a prewarmed 50 ml-falcon tube in a 42°C water bath. After cells were incubated at this temperature for 10, 20 and 30 minutes, GFP fluorescence and cell density was determined. Cells treated with 5 µM TPEN were used as positive control. Error bars represent standard deviation (SD). (TIF) [file pone.0025136.s001.tif]

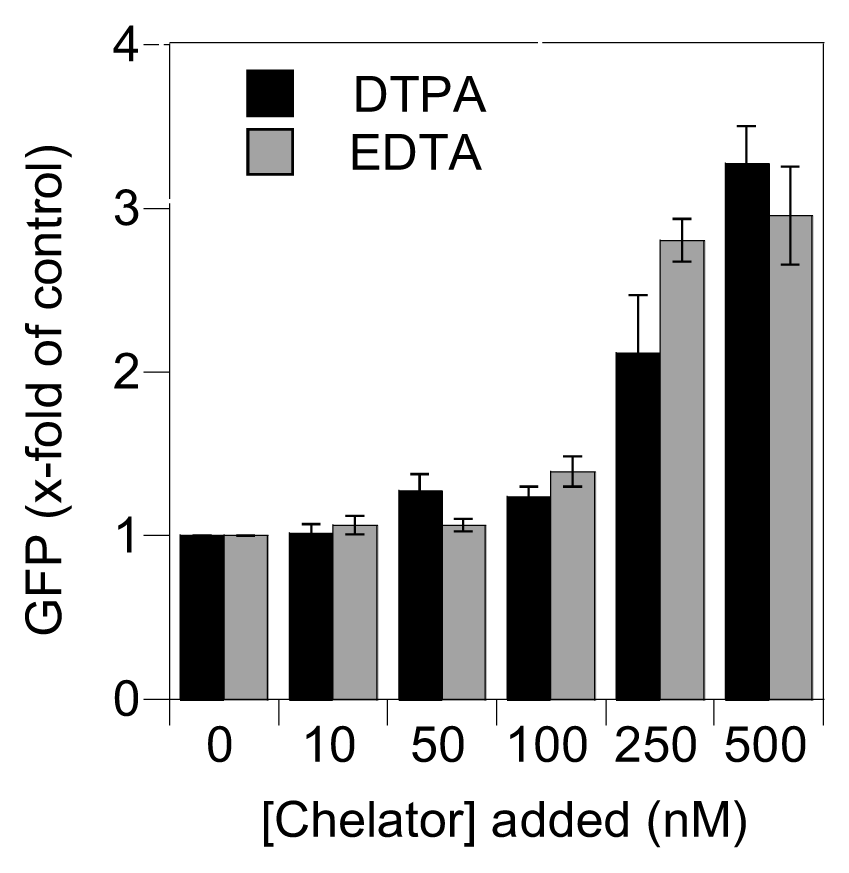

Supplement: Figure S2 — Dose response of GFP fluorescence after treatment with DTPA and EDTA. Cells transformed with the ZRE-GFP promoter-reporter construct were grown in RPMI-1640 medium with indicated chelator concentrations for 20 hours at 30°C. Fluorescence signal was normalized by cell density and is expressed as x-fold increase of untreated sample. Error bars represent standard deviation (SD). (TIF) [file pone.0025136.s002.tif]

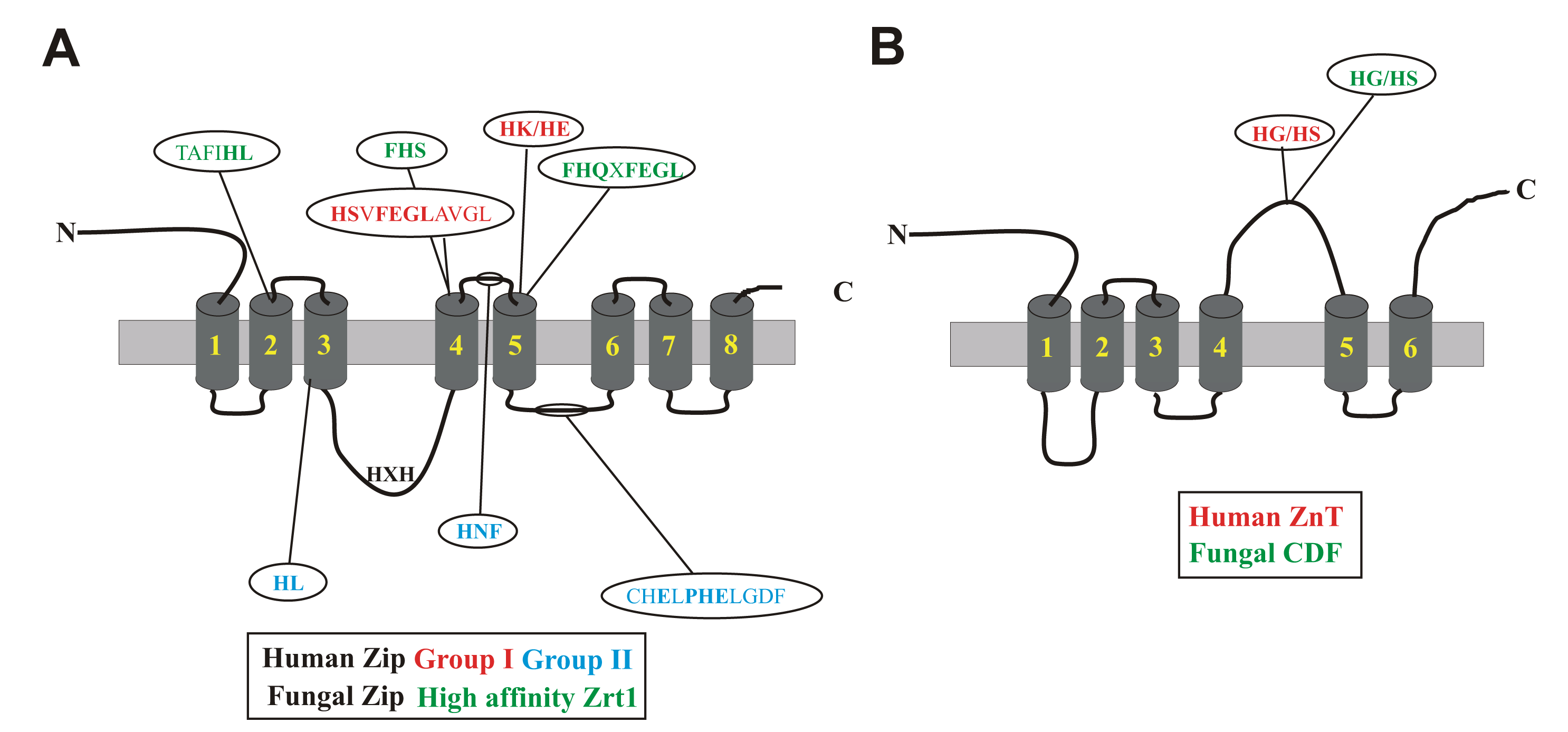

Supplement: Figure S3 — Motif comparison of Zinc transporters. Human and fungal zinc transporters of the Zip family (A) and CDF family (B) were analyzed for conserved functional motifs. Motifs of human CDF and Zip group I transporter are shown in red, hZip group II in blue and fungal transporters in green. TMpred and ClustalW2 were used for motif analysis. (TIF) [file pone.0025136.s003.tif]
