# Supplementary material for: High-Throughput Screen for Identifying Small Molecules That Target Fungal Zinc Homeostasis
Source: PLoS One. 2011 Sep 29;6(9):e25136. doi: 10.1371/journal.pone.0025136 (PMC3182986; doi:10.1371/journal.pone.0025136)
Supplement: Table S1 — Summary of Hit Compounds. (PDF) [file pone.0025136.s004.pdf]

Table S1 Summary of Hit Compounds

Group	Category	drug name	% induction
A	2	BENZETHONIUM CHLORIDE	76
A	2	CLOFAZIMINE	75
A	2	CHLORHEXIDINE	74
A	2	ALEXIDINE HYDROCHLORIDE	71
A	2	CHLOROXINE	66
A	2	METHYLBENZETHONIUM CHLORIDE	65
A	2	RITANSERIN	65
A	2	CLIOQUINOL	60
A	2	ETHACRIDINE LACTATE	59
A	2	PERHEXILINE MALEATE	58
A	2	PROMAZINE HYDROCHLORIDE	57
A	2	CHLORPROMAZINE	54
A	2	CICLOPIROX OLAMINE	52
A	3	DISULFIRAM	47
A	3	DIBENZOYLMETHANE	46
A	3	CLEMASTINE	43
A	3	SERTRALINE HYDROCHLORIDE	39
A	3	TRIMEPRAZINE TARTRATE	39
A	3	TRIFLUPROMAZINE HYDROCHLORIDE	39
A	3	DYCLONINE HYDROCHLORIDE	36
A	3	HINOKITIOL	35
A	3	CETYLPYRIDINIUM CHLORIDE	34
A	3	SANGUINARINE SULFATE	34
A	3	TRIADIMEFON	32
A	3	AMINOETHOXYDIPHENYLBORANE	31
B	1	BROMHEXINE HYDROCHLORIDE	147
B	1	2-BENZOYL-5-METHOXYBENZOQUINONE	124
B	1	FLUNARIZINE HYDROCHLORIDE	99
B	1	TERBINAFINE HYDROCHLORIDE	96
B	1	CINNARAZINE	85
B	1	ESTRADIOL ACETATE	84
B	2	MECLIZINE HYDROCHLORIDE	77
B	2	ATOVAQUONE	74
B	2	TOLNAFTATE	74
B	2	CLOPIDOGREL SULFATE	67
B	2	ZIDOVUDINE [AZT]	66
B	2	AMIODARONE HYDROCHLORIDE	65
B	2	FLOXURIDINE	62
B	2	CLARITHROMYCIN	62
B	2	TELITHROMYCIN	61

Group	Category	drug name	% induction
B	2	ROXITHROMYCIN	60
B	2	CHLOROGUANIDE HYDROCHLORIDE	60
B	2	CORALYNE CHLORIDE	59
B	2	FLUTAMIDE	59
B	2	TRISODIUM ETHYLENEDIAMINE TETRACETATE	59
B	2	NIMODIPINE	58
B	2	AMITRIPTYLINE HYDROCHLORIDE	56
B	2	ERYTHROMYCIN	55
B	2	ERYTHROMYCIN ETHYLSUCCINATE	55
B	2	HYGROMYCIN B	55
B	2	SIMVASTATIN	53
B	2	HEPTACHLOR	51
B	3	DIURON	50
B	3	ZOXAZOLAMINE	49
B	3	ANTIMYCIN A	47
B	3	TOTAROL-19-CARBOXYLIC ACID, METHYL ESTER	47
B	3	GUAIOL(-)	47
B	3	METHOXYCHLOR	45
B	3	CLINDAMYCIN HYDROCHLORIDE	44
B	3	DICHLORODIPHENYLDICHLOROETHYLENE	42
B	3	DIMERCAPROL	41
B	3	PHENOXYBENZAMINE HYDROCHLORIDE	41
B	3	PHENELZINE SULFATE	41
B	3	ARTENIMOL	41
B	3	PYRIDOSTIGMINE BROMIDE	40
B	3	ARIPIPRAZOLE	40
B	3	AMBROXOL HYDROCHLORIDE	40
B	3	ETHOPROPAZINE HYDROCHLORIDE	40
B	3	BLASTICIDIN S	39
B	3	VALINOMYCIN	39
B	3	TRIHEXYPHENIDYL HYDROCHLORIDE	38
B	3	PROADIFEN HYDROCHLORIDE	38
B	3	DOXEPIN HYDROCHLORIDE	38
B	3	OXETHAZAINE	38
B	3	PHTHALYSULFATHIAZOLE	38
B	3	DIBENZOTHIOPHENE	38
B	3	RILUZOLE	38
B	3	COLISTIN SULFATE	37
B	3	NEROLIDOL	37
B	3	OSAJIN	37
B	3	CRYPTOTANSHINONE	37

Group	Category	drug name	% induction
B	3	2',beta-DIHYDROXYCHALCONE	37
B	3	ESTRADIOL PROPIONATE	37
B	3	PALMATINE CHLORIDE	36
B	3	CEDRELONE	36
B	3	NICLOSAMIDE	36
B	3	BEPRIDIL HYDROCHLORIDE	35
B	3	ALAPROCLATE	35
B	3	AZITHROMYCIN	35
B	3	CRINAMINE	35
B	3	VINPOCETINE	34
B	3	TETRACAINE HYDROCHLORIDE	33
B	3	TOLTRAZURIL	33
B	3	AMILORIDE HYDROCHLORIDE	33
B	3	BENSERAZIDE HYDROCHLORIDE	32
B	3	BERGAPTEN	32
B	3	ORPHENADRINE CITRATE	32
B	3	NICERGOLINE	31
B	3	SIBUTRAMINE HYDROCHLORIDE	31
B	3	CLEMIZOLE HYDROCHLORIDE	30
B	3	DEHYDROABIETAMIDE	30
B	3	TANSHINONE IIA	30
B	3	TETRANDRINE	30
