# Supplementary material for: High-Throughput Screen for Identifying Small Molecules That Target Fungal Zinc Homeostasis
Source: PLoS One. 2011 Sep 29;6(9):e25136. doi: 10.1371/journal.pone.0025136 (PMC3182986; doi:10.1371/journal.pone.0025136)
Supplement: Table S2 — Protein sequence comparison between human and fungal A) Zip and B) CDF transporters. (PDF) [file pone.0025136.s005.pdf]

Table S2 Protein sequence comparison between human and fungal A) Zip and B) CDF transporters.

A

	**hZip***													
	**1**	**2**	**3**	**4**	**5**	**6**	**7**	**8**	**9**	**10**	**11**	**12**	**13**	**14**
**ScZrt1**[1)]	13	16	19	10	7	9	11	8	8	9	10	11	12	9
**ScZrt2**[1)]	14	17	19	10	10	12	6	8	14	7	14	9	11	12
**CaZrt1**[2)]	12	17	12	8	7	8	11	6	15	13	9	14	9	7
**CaZrt2**[2)]	16	18	18	14	14	13	8	7	12	11	13	10	9	12
**AfZrfB**[3)]	16	22	16	9	15	12	5	11	10	8	16	7	8	7
**AfZrfC**[3)]	16	19	20	10	15	10	17	10	12	11	12	12	15	7

B

	**hZnT***								
	**1**	**2**	**3**	**4**	**5**	**6**	**7**	**8**	**9**
**ScZrc1**[1)]	27	22	27	21	22	16	31	24	9
**ScCot1**[1)]	28	19	21	16	20	12	23	18	9
**ScZrg17**[1)]	5	5	7	7	15	11	9	11	7
**ScMsc2**[1)]	2	12	14	16	7	10	7	12	6

[1)]Saccharomyces cerevisiae protein sequence obtained from http://www.yeastgenome.org
[2)]Candida albicans protein sequences obtained from http://www.candidagenome.org
[3)]Aspergillus fumigatus protein sequences obtained from http://www.aspgd.org
* Human protein sequences obtained from http://www.uniprot.org/uniprot
Multiple sequence analysis has been done in ClustalW2
